# A Low-Voltage Low-Power Voltage-to-Current Converter with Low Temperature Coefficient Design Awareness

**DOI:** 10.3390/s25041204

**Published:** 2025-02-16

**Authors:** Haoze Chen, Pak Kwong Chan

**Affiliations:** School of Electrical and Electronics Engineering, Nanyang Technological University, Singapore 639798, Singapore; chen1640@e.ntu.edu.sg

**Keywords:** low voltage, voltage-to-current converter, three-stage operational transconductance amplifier, temperature coefficient, sensor signal processing

## Abstract

This paper presents a low-voltage, low-power voltage-to-current converter (V-I Converter) implemented in TSMC 40 nm CMOS technology. Operating at a supply voltage of 0.45 V with an input range of 0.1 V to 0.3 V, the proposed circuit achieves a temperature coefficient of 54.68 ppm/°C, which is at least 2× better than prior works, ensuring stable performance across a wide temperature range (−20 °C to 80 °C). The design employs a three-stage operational transconductance amplifier (OTA) with a Q-reduction frequency compensation technique to produce programmable output currents while maintaining a power dissipation of less than 2.76 μW. With a bandwidth of 34.45 kHz and a total harmonic distortion (THD) of −56.66 dB at 1 kHz and 0.1 V_PP_ input signal, the circuit demonstrates high linearity and low power consumption under ultra-low voltage design scenarios. These features make the proposed V-I Converter highly suitable for energy-constrained applications such as biomedical sensors, energy harvesting systems, and IoT nodes, where low power consumption and temperature stability are critical parameters.

## 1. Introduction

As research advances in the areas of microsensors, biomedical implantable devices, portable electronic equipment, and other applications where speed is not of concern, there is an increasing demand for ultra-low voltage and low power consumption. Furthermore, the continuous down-scaling of the process results in the gate oxide becoming thinner. It is then more susceptible to breakdown, so the supply voltage needs to drop to ensure device stability [1]. To meet this demand, designing circuits with MOS transistors operating in the subthreshold region has emerged as a widespread approach since the mid-1970s [2]. Notably, the subthreshold CMOS circuits offer a high gm/ID ratio and exceptional energy efficiency [3].

The V-I Converter converts voltage signals to current signals, and this becomes a significant analog signal-processing block for different usages in integrated circuits and systems. Based on the function of the V-I Converter, a Gm-C filter, a data converter, a multiplier and a voltage-to-frequency converter, which are the basic circuits for use in sensors, can be built [4,5,6,7]. For biomedical sensors, the V-I Converter can be applied for the analog pre-processing of very low biomedical signals. It is also suitable for use as a low-frequency analog front-end for biomedical sensor interfaces [8]. In terms of portable devices, the V-I Converter adjusts the output current by feeding back current to the input voltage of a hearing aid device [9,10]. In a MEMS gyroscope, the V-I converter is used to precisely and dynamically adjust the temperature of the heater [11]. In addition, in the application of a VCO-based sensor, a voltage-to-current converter is needed to generate the feedback current in order to achieve a small noise variation at the output of the VCO [12]. Therefore, the V-I Converter can be considered a common basic block for sensor applications.

The V-I Converter has a universal expression, as follows:

*I_out_ = K∙V_in_,* (1)

where *I_out_* is the output current, *V_in_* is the input voltage and *K* is a constant.

In the typical design of a V-I Converter, designers focus more on linearity. Turning to very low-voltage applications, circuit designers also seek low power consumption. On top of that, trade-off parameters like bandwidth (BW), power supply rejection ratio (PSRR), common mode rejection ratio (CMRR), input-referred noise, total harmonic distortion (THD), rail-to-rail input range, and power consumption are also addressed. In previous research, temperature stability has been given little consideration. In very low-voltage circuits, temperature drift can have a significant effect on the precision. For example, the variation arising from temperature drift may be fed back or amplified and processed within the sensor system, resulting in an error in the output of the sensor. Temperature instability is particularly fatal in low-frequency, low-voltage sensor circuits because it consumes the available headroom under a limited supply voltage.

This raises the motivation of this work, which aims to design a very low-voltage voltage-to-current converter with design awareness for achieving a low temperature coefficient. In order to prepare the V-I Converter for application in a variety of scenarios, the output current is made programmable so as to yield flexibility and meet different requirements in terms of precision in the context of process variation. The following section of this paper is organized as follows. Section 2 reviews the previous design of the V-I Converter. Section 3 describes the design of the proposed 0.45 V V-I Converter with temperature-compensated output currents. Section 4 presents the simulation results and discussions. Section 5 gives the conclusion.

## 2. Review of V-I Converters

**Figure 1:**
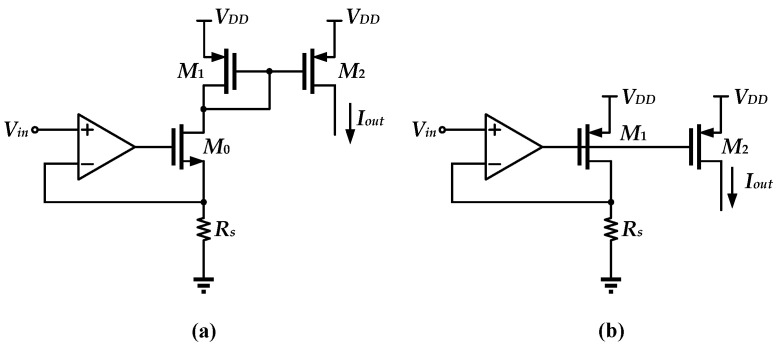
Conventional V-I structures: (**a**) Using NMOS as the driving transistor; (**b**) using PMOS as the driving transistor.

A traditional voltage-to-current circuit is implemented by OTA, as shown in Figure 1. In this conventional structure, OTA always drives a MOSFET and the resistor in a negative feedback loop [13]. Negative feedback can make the negative input of the OTA match closely to the output voltage, achieving an output current approximately equal to *V_in_*/*R_S_*. In this topology, the driving transistor is either an NMOS or a PMOS. As shown in Figure 1a, the NMOS is used as the driving transistor, and the output current is copied through the PMOS current mirror. The minimum supply voltage is about *V_in_*_,*max*_ *+ V_DS_*_0_ *+ V_SG_*_1_. Instead, the supply voltage can be further reduced to *V_in_*_,*max*_ *+ V_SD_*_1_ if the PMOS is used as the driver transistor, as shown in Figure 1b.

**Figure 2:**
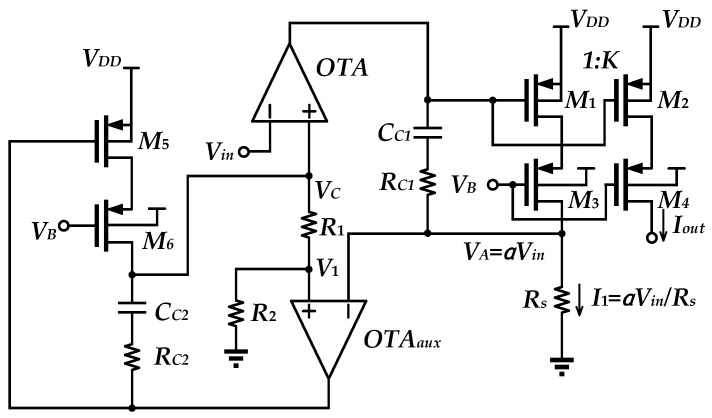
FBVA [7].

The topology shown in Figure 1b is extended to another improved topology (Figure 2) [7], which is called the feedback voltage-attenuated (FBVA) V-I Converter.

In the FBVA structure, the OTA permits *V_C_* = *V_in_* under a high-gain feedback loop. With voltage dividing the function according to the feedback resistors *R*_1_ and *R*_2_ of the OTA_aux_-*M*_1_*-M*_4_ feedback loop, *V_A_* becomes *αV_in_*, where *α* = *R*_2_*/*(*R*_1_ *+ R*_2_). Then, the output current will become *I_out_* = *αV_in_∙K/R_s_*. As *C_C_*_2_ and *R_C_*_2_ create a zero to cancel a pole, this permits the FBVA V-I Converter to extend the bandwidth. Through the attenuation provided by the negative feedback loop, the FBVA V-I Converter displays low distortion levels. Despite this, this topology achieves improved temperature compensation over that of the conventional V-I Converter. However, this structure, making use of multiple OTAs, increases the complexity. Of particularly note, since it uses a cascade of transistors, it leads to an increase in supply voltage requirements.

**Figure 3:**
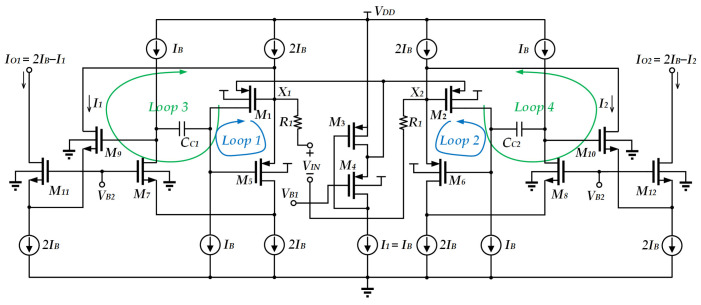
V-I Converter with nested feedback loops [14].

Another reported circuit [14] consists of a differential pair with two nested feedback loops, as depicted in Figure 3. The two main loops (loop 1 and loop 2) consist of *M*_1_ and *M*_2_ and *M*_5_ and *M*_6_*,* used for reducing the input impedance through negative feedback. *M*_3_, *M*_4_ and the current source *I*_1_ comprise a flipped voltage follower, which is used to reduce the source impedance of *M*_4_, *M*_1_, and *M*_2_ such that the voltages of node X_1_ and node X_2_ are close to *V_B_*_1_. This meets the condition of passing equal current. The third loop, which consists of *M*_5_, *M*_7_, and *M*_9_, and the fourth loop, which consists of *M*_6_, *M*_8,_ and *M*_10_, are the high-gain negative feedback loops. They further reduce the impedance of nodes X_1_ and X_2_ whilst transferring the input current to the output without replication. Besides this, the high-gain feedback loop helps reduce the mismatch between each pair of transistors. Overall, the circuit achieves a high degree of linearity for voltage-to-current conversion. However, this circuit still relies on high supply voltage.

**Figure 4:**
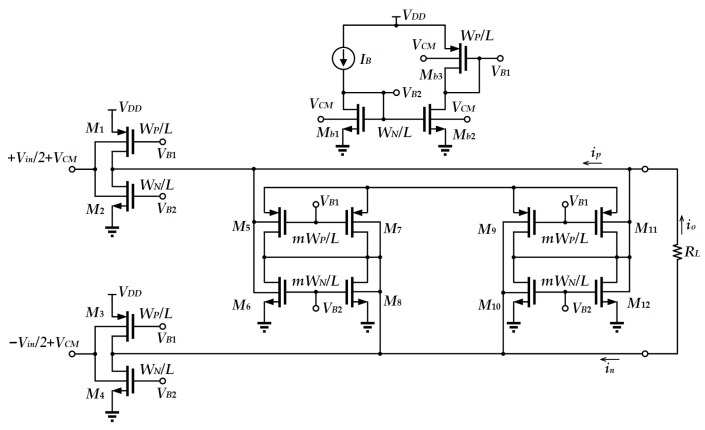
Bulk-driven transconductor.

To allow very low-voltage operation, a bulk-driven CMOS inverter-based tunable transconductor has been proposed [15]. As depicted in Figure 4, *M*_5_–*M*_12_ form a positive feedback loop, which aims to provide a relatively large DC gain. All transistors operating in the subthreshold region permit the circuit to operate at a voltage as low as 0.25 V. Through the subthreshold current equation and the threshold voltage equation [15], the output current is derived as

(2) $$i_{o}=i_{p}-i_{n}\approx \frac{I_{B}}{2V_{T}}\left(\frac{\gamma_{p}}{2n_{p}\sqrt{2\left|\phi_{Fp}\right|-\left(V_{DD}-V_{CM}\right)}}+\frac{\gamma_{n}}{2n_{n}\sqrt{2\left|\phi_{Fn}\right|-V_{CM}}}\right)V_{in}.$$

From (2), the overall transconductance of the proposed circuit is obtained as $\frac{I_{B}}{2V_{T}}\left(\frac{\gamma_{p}}{2n_{p}\sqrt{2\left|\phi_{Fp}\right|-\left(V_{DD}-V_{CM}\right)}}+\frac{\gamma_{n}}{2n_{n}\sqrt{2\left|\phi_{Fn}\right|-V_{CM}}}\right)$.

The advantage of this design is that it provides a good trade-off, offering ultra-low supply voltage with good linearity whilst providing tunable transconductance. However, the bulk-driven transistors also suffer from several disadvantages, such as the use of the triple-well CMOS technology in realization [15], a higher input noise, a higher offset voltage [16] and higher fabrication costs when compared to the single-well CMOS technology. Moreover, the substrate channel PN junction is likely to latch-up in cases of not being properly designed, causing reliability issues.

**Figure 5:**
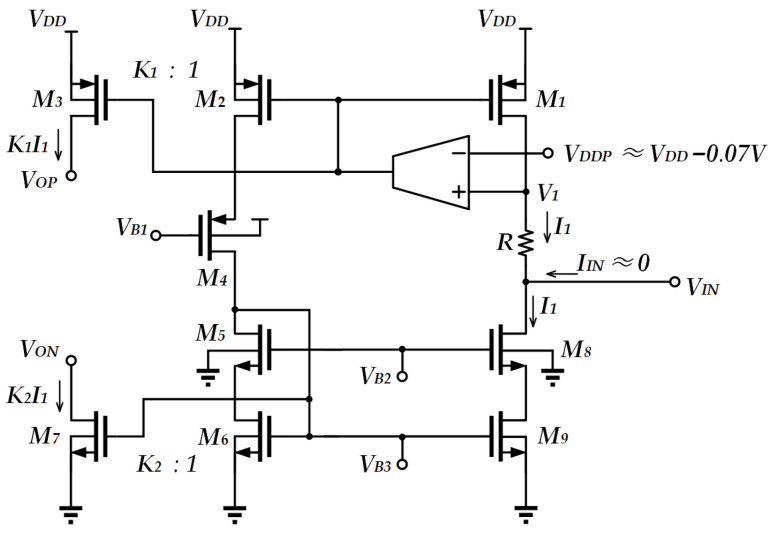
Low-voltage linear V-I conversion unit [17].

In order to avoid the undesirable effects of bulk driving, another reported work [17] focused on gate driving as shown in Figure 5. Due to the larger transconductance of the device, this circuit shows better noise performance when compared to that of bulk-driven transistors. It also optimizes the swing range of the output voltage. The circuit also generates sinking and sourcing currents and exhibits a large input common-mode voltage range. However, the current *I*_1_ = (*V_DDP_* − *V_IN_*)/*R* may be subject to the offset arising from the matching between the sourcing current and the sinking current. The input impedance of the circuit depends on the effective output impedance of the current mirror.

## 3. Proposed V-I Converter

### 3.1. V-I Converter Architecture

**Figure 6:**
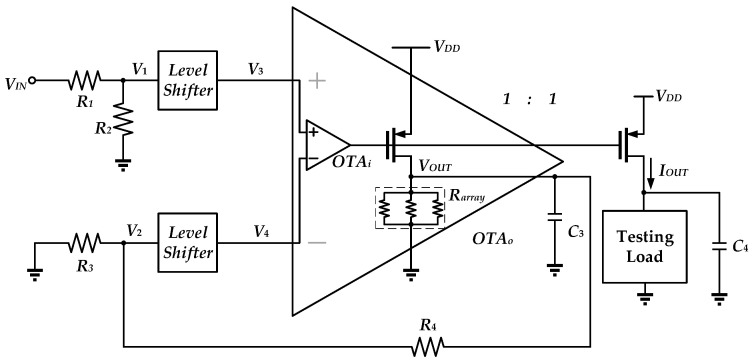
Topology of proposed circuit architecture.

Figure 6 illustrates the topology of the proposed circuit. The circuit operates in the subthreshold region. This circuit consists of peripheral resistors, level shifters, and the OTA_o_. The resistors *R*_1_–*R*_4_ are formed by PMOS devices, which have small sizes and are known as pseudo-resistor (PR) devices [18]. These PR devices display very high impedance properties so that there is no loading effect on the input source and the output stage of OTA_o_. The function of the level shifter is to raise the input voltages *V*_1_ and *V*_2_ to higher voltages *V*_3_ and *V*_4_ in order to satisfy the input voltage range of the OTA_o_ and to produce an intentional temperature coefficient in the circuit. The OTA_o_ consists of an OTA_i_, which consists of two stages with active loads and a third stage formed by the PMOS driver transistor that drives the resistor array with the temperature compensation. The output current will be *I_OUT_* = *V_OUT_/R_array_*. With *R*_1_ = *R*_2_ = *R*_3_ = *R*_4_ = *R*, *V_OUT_* = *V_IN_*. By choosing *R*_1_ = *R*_2_ = *R*_3_ = *R*_4_, a symmetrical circuit structure can be procued, which offers a good common mode rejection ratio.

#### 3.1.1. Design of Pseudo Resistor (PR)

**Figure 7:**
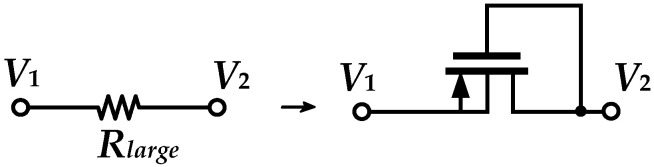
PR.

PR, which was introduced by Delbrück in 1984 [18], usually uses a PMOS transistor as a diode connection to operate in the cutoff region to provide an extremely large resistance. It is well-known and widely used for the acquisition of bioelectrical signals [19,20,21]. For the connection shown in Figure 7, if *V*_1_ is greater than *V*_2_, this structure is equivalent to a PMOS using a diode connection. If *V*_1_ is less than *V*_2_, the structure behaves like a bipolar transistor due to the positively biased PN junction formed by the drain of PMOS and the substrate. In this design, *V*_1_ is greater than *V*_2_, with *V*_1_ − *V*_2_ *< |V_THP_|*, to allow PR to be in the cutoff region.

**Figure 8:**
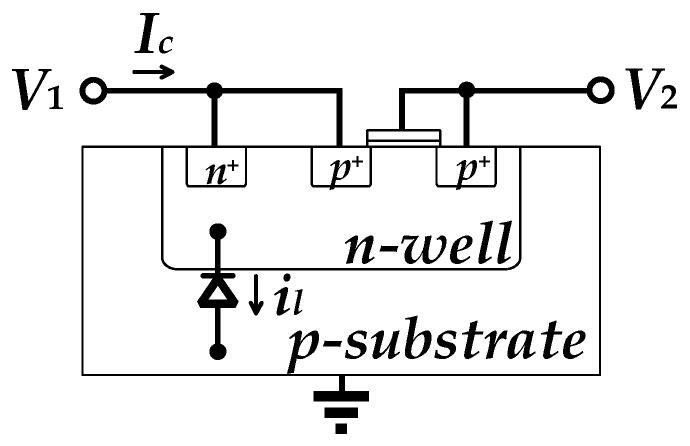
Cross-section of the PR.

PR has an exponential I-V characteristic, and therefore poor linearity and a strong dependence on temperature and process. Another limitation of PR is the problem of current leakage due to parasitic effects. In diode-connected PRs, intrinsic leakage currents can exist in the diode formed between the p-substrate and the n-well of the PMOS transistor, thus affecting the output offset of the OTA [22]. As shown in Figure 8, *I_c_* represents the current during normal conduction, whereas *i_l_* represents the reverse leakage current of the parasitic diode. If *V*_1_ is connected to the high voltage terminal and *V*_2_ is connected to the low voltage terminal, an extra voltage drop is generated by *i_l_* due to the leakage current in the reverse-biased diode. This phenomenon causes the mismatch at the two input ports of the OTA, leading to an output DC voltage offset. This problem will be further pronounced if the circuit features high gain. In order to suppress such a DC offset, the symmetric topology, depicted in Figure 6, is utilized to match the resistances in the positive and negative branches of the OTA. As such, the leakage current is treated as the common-mode signal and is rejected by the converter in a form amplifier topology.

#### 3.1.2. Design of Level Shifter

**Figure 9:**
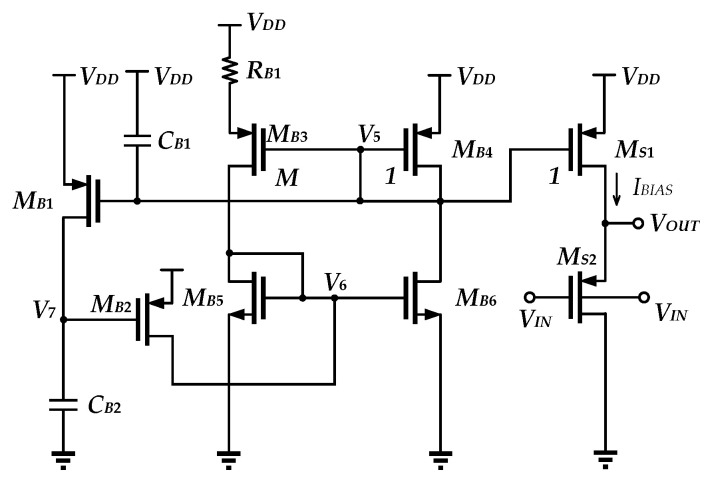
The level shifter.

The level shifter depicted in Figure 9 contains a startup circuit that comprises *M_B_*_1_, *M_B_*_2_, *C_B_*_1_, and *C_B_*_2_, a bias circuit that comprises *M_B_*_3_–*M_B_*_6_, *R_B_*_1_, and a source follower formed by *M_S_*_1_ and *M_S_*_2_.

The bias current *I_BIAS_* is calculated as

(3) $$I_{BIAS}=\frac{nV_{T}In\left(M\right)}{R_{B1}},$$

where *M* denotes the ratio of aspect ratio of *M_B_*_3_ with respect to that of *M_B_*_4_ and *M_B_*_1_.

When the power is on, *V*_5_ rises to *V_DD_*, forcing *M_B_*_1_ to turn on and causing the ac shorting *V*_7_ to *GND*, thus making *M_B_*_2_ on. As a result, *V*_6_ is pulled high, which in turn causes the bias circuit to generate the bias current. *V*_7_ is then pulled high due to *M_B_*_1_’s operation, which causes *M_B_*_2_ to be turned off. *V*_5_ is pulled low after the bias current has been established. Then, *M_B_*_1_ is turned off. When the capacitive-based startup circuit has completed its action, it is isolated and no longer contributes any additional current, hence reducing power consumption.

*M_S_*_2_ and *M_S_*_1_ form a source follower with the gate-bulk driving transistor *M_S_*_2_. To raise identical voltage potential, the PMOS with gate-bulk connection uses a smaller size when compared to the PMOS with source-bulk connection. It is observed that the input-referred noise decreases with the increase in bias current in the level shifter. Although it permits lower input-referred noise, it degrades the temperature coefficient. On the contrary, the gate-bulk-connected PMOS can optimize such a trade-off. This is mainly because of the change in threshold voltage, which leads to a change in temperature coefficient, which favors the temperature compensation case.

#### 3.1.3. Design of OTA_o_

**Figure 10:**
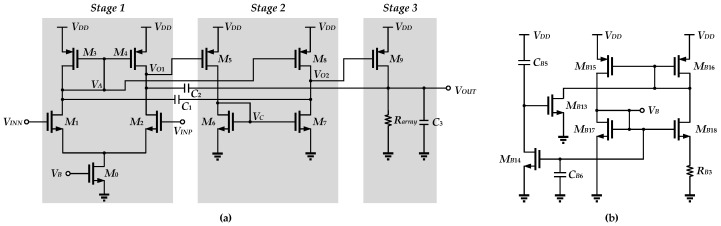
(**a**) The design of the OTA_o_ with resistor array as the passive load in the output stage; (**b**) the bias circuit.

Due to the low supply voltage, the OTA can only operate in the subthreshold region, and the *V_DS_* needs to be greater than 100 mV in order to allow the MOSFETs to operate in a valid region. Besides this, the use of a cascode structure to increase the DC gain is not permitted under ultra-low-voltage design. Although the two-stage topology on the basis of a single Miller compensation capacitor is simple and efficient in terms of size, the overall DC gain may be not sufficient to meet precision requirements due to the intrinsic low output impedance of CMOS devices in the 40 nm technology node. Therefore, in order to obtain an OTA with a large gain whilst operating in the subthreshold region, this OTA design is based on the modification of the amplifier structure [23], as shown in Figure 10a. The main difference is that of the passive temperature compensation resistor array replacing the active load in this design of the final output stage.

**Figure 11:**
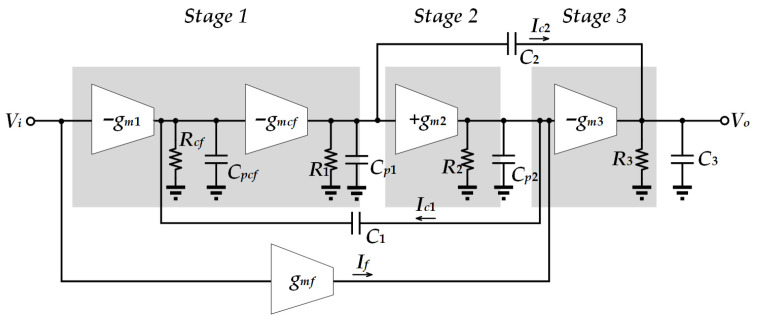
System block of OTA_o_.

The system block diagram is shown in Figure 11, where *g_m_*_1_ is the transconductance of the first stage, contributed by *M*_1_. *g_mcf_* is the transconductance of *M*_3_. *g_m_*_2_ is the transconductance of the second stage, contributed by *M*_5_. *g_m_*_3_ is the transconductance of the third stage, contributed by *M*_9_. *g_mf_* is the transconductance of the feedforward path, used to generate the left half-plane (LHP) zero, contributed by *M*_1_, *M*_3_, and *M*_8_. *R_cf_* is the effective output resistance of *M*_1_ and is approximately equal to 1/*g_mcf_*. *R*_1_ is the output resistance of the first stage. *R*_2_ is the output resistance of the second stage. *R*_3_ is the output resistance of the third stage, consisting of *R_array_* and the equivalent output impedance of *M*_9_ in parallel. *C_pcf_* is the equivalent capacitance at the output of *M*_1_. *C_p_*_1_ is the output capacitance of the first stage. *C_p_*_2_ is the output capacitance of the second stage. *C*_3_ is the output capacitance of the third stage. *C*_1_, together with *M*_3_ and *M*_4_, forms a current buffer to reduce the potential large Q generated by the non-dominant pole, thus slowing down the sharp changes in phase. *C*_2_ is a Miller capacitor used to achieve pole splitting and increase the phase margin.

To analyze the loop stability of this OTA_o_, the following assumptions are made: (1) *C*_1_, *C*_2_, and *C*_3_ are larger than the internal capacitances of the circuit; (2) *g_m_*_3_ is larger than *g_m_*_1_, *g_mcf_*, and *g_m_*_2_; and (3) the gain of each gain stage (*g_m_*_1_*R*_1_, *g_m_*_2_*R*_2_, and *g_m_*_3_*R*_3_) > 1, except for *g_mcf_R_cf_ ≈* 1. Thus the transfer function can be approximated as follows:

(4) $$H\left(s\right)=\frac{-A_{DC}\cdot \left(1+s\frac{C_{2}g_{mf}}{g_{m1}g_{m2}}\right)}{\left(1+p_{1}s\right)\left[1+s\cdot \frac{C_{1}\left(C_{3}+C_{2}g_{m3}R_{cf}\right)}{C_{2}g_{m3}}+\frac{\left(C_{p2}+C_{1}\right)C_{3}}{g_{m2}g_{m3}}s^{2}\right]},$$

where the *DC* gain is *A_DC_* = *g_m_*_1_*g_m_*_2_*g_m_*_3_*g_mcf_R*_1_*R*_2_*R*_3_*R_cf_ ≈ g_m_*_1_*g_m_*_2_*g_m_*_3_*R*_1_*R*_2_*R*_3_, and the dominant pole is *p*_1_ = 1/(*C*_2_*g_m_*_2_*g_m_*_3_*R*_1_*R*_2_*R*_3_). Thus the gain-bandwidth product (GBW) is obtained as

(5) $$GBW=A_{DC}\cdot p_{1}=\frac{g_{m1}}{C_{2}}$$

From (4), this OTA_o_ yields a zero, as

(6) $$z_{1}=\frac{g_{m1}g_{m2}}{C_{2}g_{mf}}$$

which is designed for phase lead such that the circuit derives a better phase margin. The relationship in the denominator of the transfer function *H*(*s*) is

(7) $${\left[\frac{C_{1}\left(C_{3}+C_{2}g_{m3}R_{cf}\right)}{C_{2}g_{m3}}\right]}^{2}\leq 4\frac{\left(C_{p2}+C_{1}\right)C_{3}}{g_{m2}g_{m3}}.$$

The pair of complex poles becomes

(8) $$\begin{array}{cc} w_{O} & =\left|\frac{-\frac{C_{1}\left(C_{3}+C_{2}g_{m3}R_{cf}\right)}{C_{2}g_{m3}}\pm i\cdot \sqrt{4\frac{\left(C_{p2}+C_{1}\right)C_{3}}{g_{m2}g_{m3}}-{\left[\frac{C_{1}\left(C_{3}+C_{2}g_{m3}R_{cf}\right)}{C_{2}g_{m3}}\right]}^{2}}}{2\frac{\left(C_{p2}+C_{1}\right)C_{3}}{g_{m2}g_{m3}}}\right|\\ & =\sqrt{\frac{g_{m2}g_{m3}}{\left(C_{p2}+C_{1}\right)C_{3}}} \end{array}.$$

Due to the complex conjugate poles, excessive *Q* values can occur. This causes the amplitude–frequency curve to display an overshoot effect. When this large *Q* occurs near the GBW, a sharp drop in the phase margin [24] is observed. The *Q* value is obtained as

(9) $$Q=\sqrt{\frac{C_{p2}C_{3}}{g_{m2}g_{m3}}}\cdot \frac{C_{2}g_{m3}}{C_{1}\left(C_{3}+C_{2}g_{m3}R_{cf}\right)}.$$

To reduce *Q*, it is apparent from (9) that *C*_1_, as well as *R_cf_,* needs to be increased. Besides this, the phase margin [23] is given as follows:

(10) $$\begin{array}{cc} PM & =180{}^{\circ}-\tan^{-1}\left(\frac{GBW}{p_{1}}\right)-\tan^{-1}\left\{\frac{GBW/w_{O}}{Q\left[1-{\left(GBW/w_{O}\right)}^{2}\right]}\right\}+\tan^{-1}\left(\frac{GBW}{z_{1}}\right)\\ & =90{}^{\circ}-\tan^{-1}\left(\frac{GBW/w_{O}}{Q\left[1-{\left(GBW/w_{O}\right)}^{2}\right]}\right)+\tan^{-1}\left(\frac{GBW}{z_{1}}\right) \end{array}.$$

**Figure 12:**
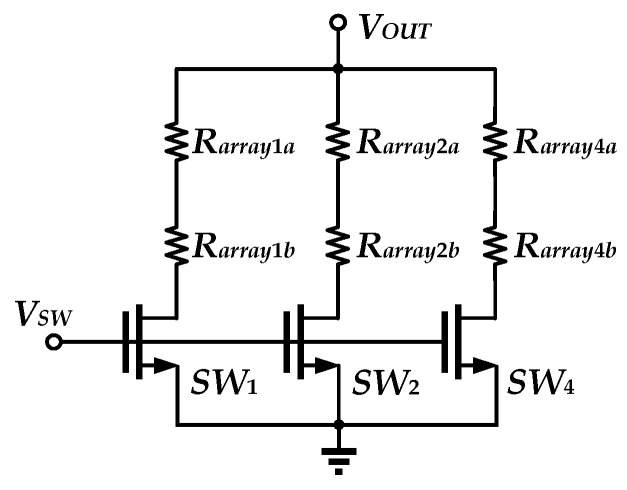
Resistor array implementation.

To achieve programmable output current, the resistor array uses a combination of three branches, as shown in Figure 12. In the process library design file, the resistor is given by

(11) $$R=R_{0}\left[1+TC_{1}\left(T-300\right)+TC_{2}{\left(T-300\right)}^{2}\right],$$

where *TC*_1_ and *TC*_2_ are the first-order temperature coefficient and the second-order temperature coefficient, respectively, and *R*_0_ is the magnitude of the resistance at the temperature of 300 K. Based on the resistor–temperature relationship, the output current needed for this circuit, the P+ poly resistor without salicide, and the P+ poly resistor with salicide are chosen for each branch of the resistor array. The types and sizes of each device of the proposed V-I Converter are shown in Table 1.

### 3.2. Temperature Compensation of Proposed V-I Converter

Temperature compensation is mainly realized by OTA_o_ and the resistor array, and in conjunction with the thermal effect contributed by the level shifter. The bias circuit is shown in Figure 10b, where *M_B_*_13_, *M_B_*_14_, *C_B_*_5_ and *C_B_*_6_ comprise the capacitive startup circuit. This constant-gm current source provides the tail current of OTA_o_, and it is given as

(12) $$I_{M0}=\frac{nV_{T}In\left[\frac{{\left(W/L\right)}_{MB18}}{{\left(W/L\right)}_{M0}}\right]}{R_{B3}}.$$

It is noted that for a P+ poly resistor without salicide, *R_B_*_3_ is given by (11), where *TC*_1_ is negative and *TC*_2_ is positive, with the relationship |*TC*_1_| >> |*TC*_2_|. Thus, *R_B_*_3_ shows the CTAT effect. Then, the derivation of *I_M_*_0_ is obtained as

(13) $$\frac{\partial I_{M0}}{\partial T}=\frac{nkIn\left[\frac{{\left(W/L\right)}_{MB18}}{{\left(W/L\right)}_{M0}}\right]}{qR_{B3}}>0,$$

It can be seen that *I_M_*_0_ is proportional to the absolute temperature (PTAT) current. *I_M_*_3_ is equal to 0.5*I_M_*_0_ and therefore also the PTAT current.

**Figure 13:**
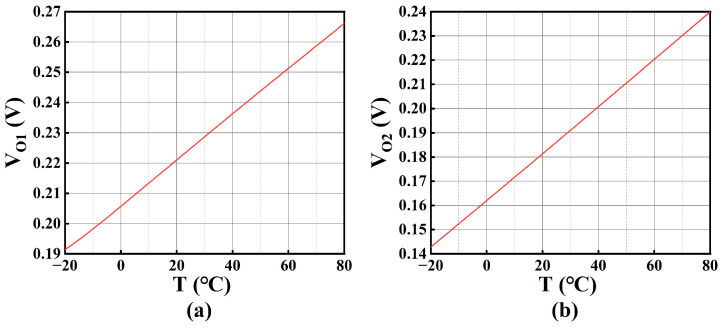
(**a**) *V_O_*_1_ versus *T*; (**b**) *V_O_*_2_ versus *T*.

The current equation of PMOS operating in the subthreshold region is

(14) $$\begin{array}{cc} I_{S} & =\mu_{p}\left(T_{0}\right){\left(\frac{T_{0}}{T}\right)}^{m_{p}}C_{ox}V_{T}^{2}\left(\frac{W}{L}\right)e^{\left(\frac{V_{SG}\left(T\right)+V_{THP}\left(T\right)}{nV_{T}}\right)}\left(1-e^{\frac{-V_{SD}\left(T\right)}{V_{T}}}\right)\left[1-\lambda V_{SD}\left(T\right)\right]\\ & \approx \mu_{p}\left(T_{0}\right){\left(\frac{T_{0}}{T}\right)}^{m_{p}}C_{ox}V_{T}^{2}\left(\frac{W}{L}\right)e^{\left(\frac{V_{SG}\left(T\right)+V_{THP\left(T\right)}}{nV_{T}}\right)}\left[1-\lambda V_{SD}\left(T\right)\right] \end{array},$$

with the electron mobility given as

(15) $$\mu_{p}\left(T\right)=\mu_{p}\left(T_{0}\right)\cdot {\left(\frac{T_{0}}{T}\right)}^{m_{p}}$$

and the threshold voltage given as

(16) $$V_{THP}\left(T\right)=V_{THP0}+\kappa_{P}\left(T-T_{0}\right)$$

where the characteristic current in weak inversion *I_S_* = *μ_p_*(*T*)*C_ox_V_T_*^2^, *V_THP_*_0_ is the threshold voltage at *T*_0_ = 300 K, *μ_p_*(*T*_0_) is the carrier mobility at *T*_0_ = 300 K, *C_ox_* is the capacitance of the gate oxide, *W* is the channel width, *L* is the channel length, *V_T_* = *kT*/*q* is the thermal voltage, *k* is Boltzmann’s constant, *q* is the electronic charge, and the subthreshold slope *n* is a constant between 1 and 3 [25,26]. *n* can be estimated to be 1.3 using this technology, which is convenient for theoretical calculation. *m_p_* is approximately 1.16, which is the exponential coefficient of *μ_p_*(*T*) with respect to temperature, and *κ_P_* is approximately 0.614 mV/K, which is the magnitude of the slope of *V_THP_*(*T*)*. m_p_* and *κ_P_* are obtained from PMOS device simulation. Since *V_S__D_* > 3 *V_T_*, this means that the *exp*[*−V_SD_*(*T*)/*V_T_*] term is small enough to be ignored, while *λ* is the channel length modulation factor. According to (14), the voltage *V_A_* in the first stage of OTA_o_ (Figure 10) is

(17) $$\begin{array}{cc} V_{A}= & V_{DD}-V_{SG3}\\ = & V_{DD}-\left\{\frac{nkT}{q}\left[In\left(\frac{I_{M3}\cdot T^{m_{p}-2}}{\mu_{p}\left(T_{0}\right)T_{0}^{m_{p}}C_{ox}{\left(k/q\right)}^{2}{\left(W/L\right)}_{M3}}\right)-\frac{q\kappa_{P}}{nk}\right]-V_{THP0,M3}+\kappa_{P}T_{0}\right\}\\ & \propto -\alpha_{1}T\left[In\left(\frac{\alpha_{2}T^{1.84}}{\alpha_{3}+\alpha_{4}TC_{1}\left(T-300\right)+\alpha_{5}TC_{2}{\left(T-300\right)}^{2}}\right)-\alpha_{6}\right] \end{array},$$

where *ɑ_n_* (*n* = 1, 2, 3…) is the lumped constant. With MATLAB (version: R2023a) verification, *V_A_* can be considered as PTAT voltage. Due to the symmetrical structure of the first stage of OTA_o_, *V_O_*_1_ is equal to *V_A_*, which implies *V_O_*_1_ is also a PTAT voltage. Figure 13a shows the plot of *V_O_*_1_ against temperature.

This PTAT voltage couples to the input of the second stage in the OTA_o_. It generates the current through *M*_5_. Since *M*_3_ and *M*_5_ have different channel lengths, this results in different threshold voltages. According to the BSIM3v3 model [27], *V_THP_*_0_ in Equation (16) can be written as

(18) $$V_{TH0}=V_{TH0}^{\prime }+K_{1}\left(\sqrt{\phi_{s}-V_{bs}}-\sqrt{\phi_{s}}\right)-K_{2}V_{bs}+K_{1}\left(\sqrt{1+\frac{NLx}{L_{eff}}}-1\right)\sqrt{\phi_{s}}-\Delta V_{TH},$$

where *K*_1_, *K*_2_, *NLx*, and ∆*V_TH_* are parameters given in [27], *V’ TH0* is the threshold voltage at zero substrate bias, and *ϕ_s_* is the surface potential. As the channel length becomes shorter, the threshold voltage shows a greater dependence on the channel length. In this design, *M*_5_ has a smaller channel length than *M*_3_, causing the threshold voltage *V_THP_*_0,*M*5_ to be less than *V_THP_*_0,*M*3_. The current *I_M_*_5_ becomes

(19) $$\begin{array}{cc} I_{M5} & =\left[\mu_{p}\left(T_{0}\right)T_{0}^{m_{p}}C_{ox}\frac{k^{2}}{q^{2}}{\left(\frac{W}{L}\right)}_{M5}e^{\frac{q\kappa_{p}}{nk}}\right]e^{\frac{q\left(V_{DD}-V_{O1}+V_{THP0,M5}-\kappa_{P}T_{0}\right)}{nkT}}T^{2-m_{p}}\\ & =\frac{{\left(W/L\right)}_{M5}}{{\left(W/L\right)}_{M3}}\cdot I_{M3}\cdot e^{-\frac{q\kappa_{P}}{nk}}\cdot e^{\frac{q\left(V_{THP0,M5}-V_{THP0,M3}\right)}{nkT}} \end{array}.$$

In (19), *V_THP_*_0,*M*5_ − *V_THP_*_0,*M*3_ is negative, resulting in the term $e^{q\left(V_{THP0,M5}-V_{THP0,M3}\right)/\left(nkT\right)}$*,* showing a PTAT effect. Since *I_M_*_3_ also has a PTAT effect, *I_M_*_5_ becomes a PTAT current. This current passes through *M*_6_ and generates the *V_C_* in Figure 10. *V_C_* can be represented as

(20) $$\begin{array}{cc} V_{C}= & \frac{nkT}{q}\left[In\left(\frac{I_{M5}\cdot T^{m_{n}-1}}{\mu_{n}\left(T_{0}\right)T_{0}^{m_{n}}C_{ox}{\left(k/q\right)}^{2}{\left(W/L\right)}_{M6}}\right)-\frac{q\kappa_{N}}{nk}\right]+V_{THN0,M6}+\kappa_{N}T_{0}\\ & \propto \alpha_{7}\cdot T\left[In\left(\alpha_{8}\cdot I_{M5}T^{0.5}\right)-\alpha_{9}\right]+\alpha_{10} \end{array},$$

where *m_n_* is approximately 1.5, and *κ_N_* is approximately 1.589 mV/K. They are characterized by simulation in this technology. According to the analytical result, *V_C_* is also a PTAT voltage. Due to the balanced design, *V_O_*_2_ is approximately equal to *V_C_* and it displays the PTAT effect. This is verified with the plot of *V_O_*_2_ against the temperature, as illustrated in Figure 13b.

**Figure 14:**
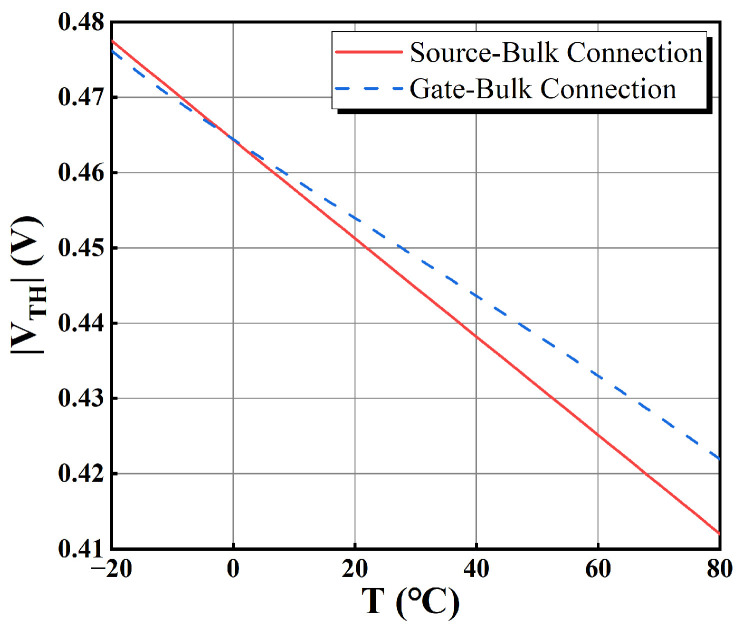
The plot of the variation of *|V_TH_|* with temperature with different bulk connections.

Assuming that the resistor array *R_array_* of the third stage has a temperature-independent property, the output current *I_M_*_9_ can be expressed as

(21) $$\begin{array}{cc} I_{M9}= & \left[\mu_{p}\left(T_{0}\right)T_{0}^{m_{p}}C_{ox}\frac{k^{2}}{q^{2}}{\left(\frac{W}{L}\right)}_{M9}e^{\frac{q\kappa_{P}}{nk}}\right]e^{\frac{q\left(V_{DD}-V_{O2}+V_{THP0,M9}-\kappa_{P}T_{0}\right)}{nkT}}T^{2-m_{p}}\\ = & \mu_{p}\left(T_{0}\right)\mu_{n}\left(T_{0}\right)T_{0}^{m_{p}+m_{n}}{C_{ox}}^{2}\frac{k^{4}}{q^{4}}{\left(\frac{W}{L}\right)}_{M9}{\left(\frac{W}{L}\right)}_{M6}e^{\frac{q\left(\kappa_{P}+\kappa_{N}\right)}{nk}}\cdot \\ & e^{\frac{q\left(V_{DD}-V_{THN0,M6}+V_{THP0,M9}-\kappa_{N}T_{0}-\kappa_{P}T_{0}\right)}{nkT}}\cdot {I_{M5}}^{-1}\cdot T^{0.34}\\ & \propto \alpha_{11}e^{\frac{\alpha_{12}}{T}}\cdot T^{0.34}\cdot {I_{M5}}^{-1} \end{array},$$

where *ɑ*_12_ is negative and it causes the term $e^{\alpha_{12}/T}$ to display a PTAT effect. Although ${I_{M5}}^{-1}$ shows a CTAT effect, the term $e^{\alpha_{12}/T}T^{0.34}$ is dominant, resulting in *I_M_*_9_ as a PTAT current. This PTAT current passes through *R_array_* and will generate the output voltage *V_OUT_* with PTAT characteristics. In order to ensure *I_M_*_9_ is temperature independent, *R_array_* needs to be positively temperature dependent so that *V_OUT_*/*R_array_* becomes temperature independent and achieves a low temperature coefficient for the output current. This can be synthesized using two types of poly resistors with opposite temperature coefficients. For the complete temperature compensation, the level shifter allows some modulation of the temperature coefficient to the output current. As depicted in Figure 9, the source follower *M_S_*_2_ uses a gate-bulk connection instead of a source-bulk connection. The use of gate-bulk connection reduces the *|V_TH_|* of PMOS and slightly modifies the trend of *|V_TH_|* with temperature, as shown in Figure 14.

Compared to PMOS with source-bulk connection, the trend of *|V_TH_|* has relatively low T.C. using the gate-bulk driven transistor in the source follower of the level shifter. According to (14), if the bias current is fixed, a more slowly decreasing *|V_TH_|* will improve the CTAT effect of the output voltage of the level shifter. This voltage entering into the OTA_o_ will cause the PTAT effect of the output voltage of OTA_i_ shown in Figure 6, which offers better temperature compensation. It allows the driven transistor to receive more CTAT compensation, which compensates for the PTAT effect brought in by the resistor array and optimizes the temperature coefficient. On the whole, the gate-bulk connection improves the operation headroom and mitigates excessive PTAT effects due to the resistor array. It makes compensation of the temperature coefficient easier.

## 4. Results and Discussions

Realized using TSMC 40 nm CMOS technology, the proposed V-I Converter circuit operates at a supply voltage of 0.45 V.

**Figure 15:**
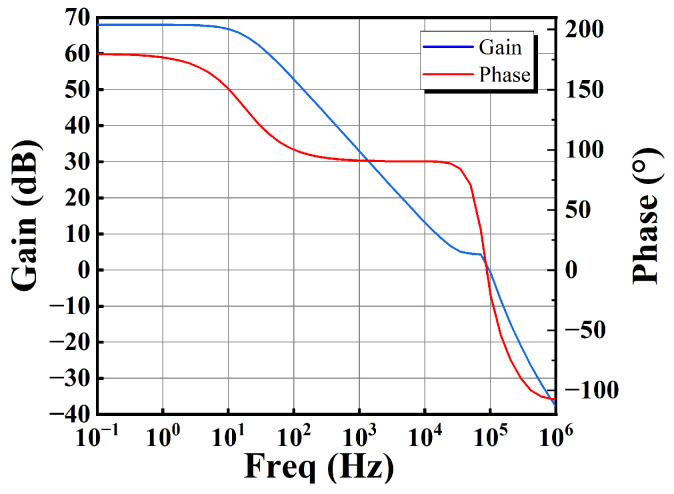
Bode plot of OTA_o_ at TT process corner.

The stability analysis of the OTA_o_ is first carried out. Its Bode plot at the TT process corner is shown in Figure 15. The DC open-loop gain is 64.79 dB, the phase margin (PM) is 80.07°, the bandwidth (BW) is 25.1 Hz, and the unity gain bandwidth (UGB) is 48.13 kHz. The stability analysis of OTA_o_, considering TT, SS, and FF process corners and fluctuations in supply voltage, is summarized in Table 2. It can be observed that the circuit sustains the function regardless of the PVT variation.

**Figure 16:**
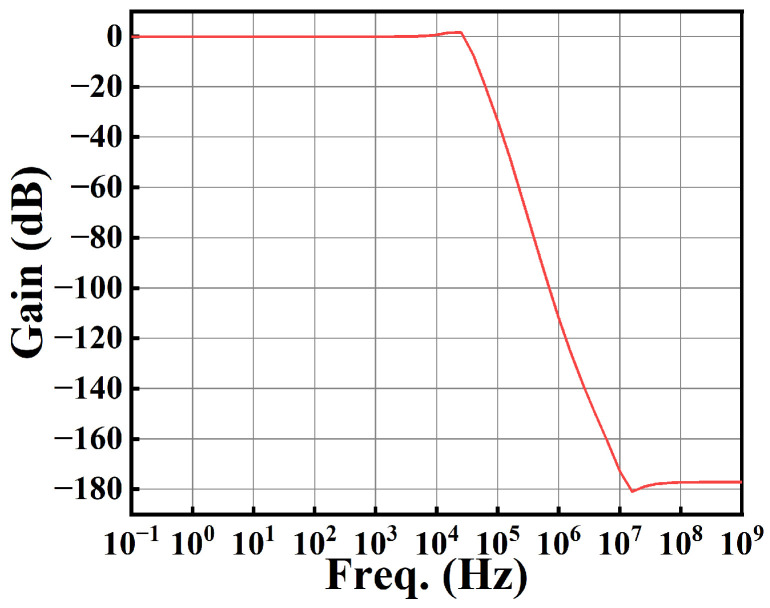
Closed-loop gain against frequency.

For the closed-loop configuration with the capacitive load capacitor *C*_3_ = 10 pF, the closed-loop gain (Figure 16) is −5.92 mdB, whereas the closed-loop bandwidth is 34.45 kHz. This indicates that the gain of the entire closed-loop system is about 1, achieving an output voltage equal to the input voltage.

**Figure 17:**
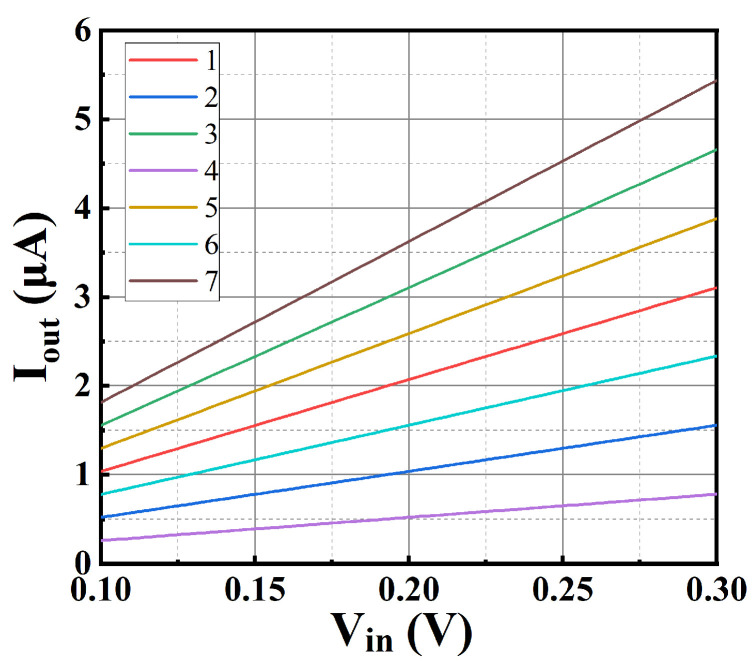
Output current versus input voltage.

The input DC voltage of the design is in the range of 0.1–0.3 V, and the programmable binary-weighted output currents at *T* = 300 K, *V_DD_* = 0.45 V and the TT process corner are shown in Figure 17. The minimum output current is 260 nA, and the maximum output current is 5.44 μA.

**Figure 18:**
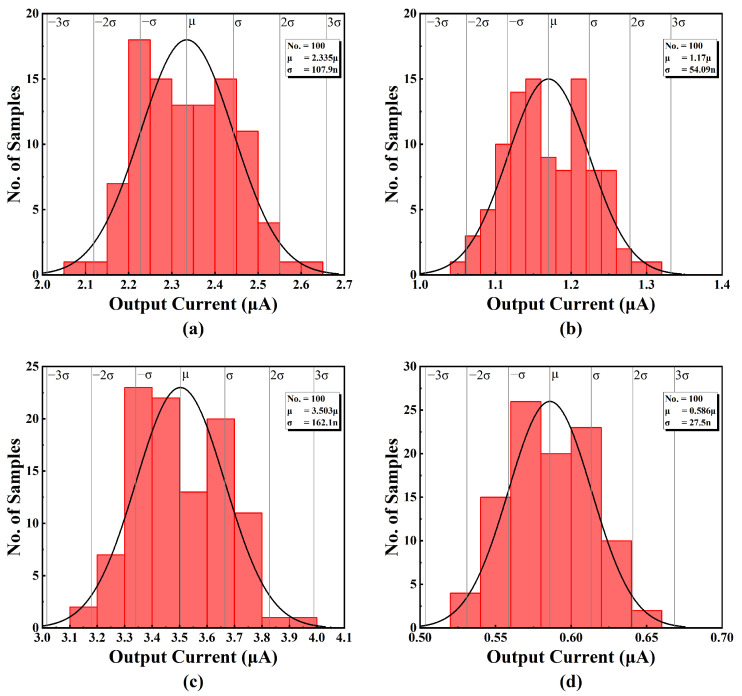

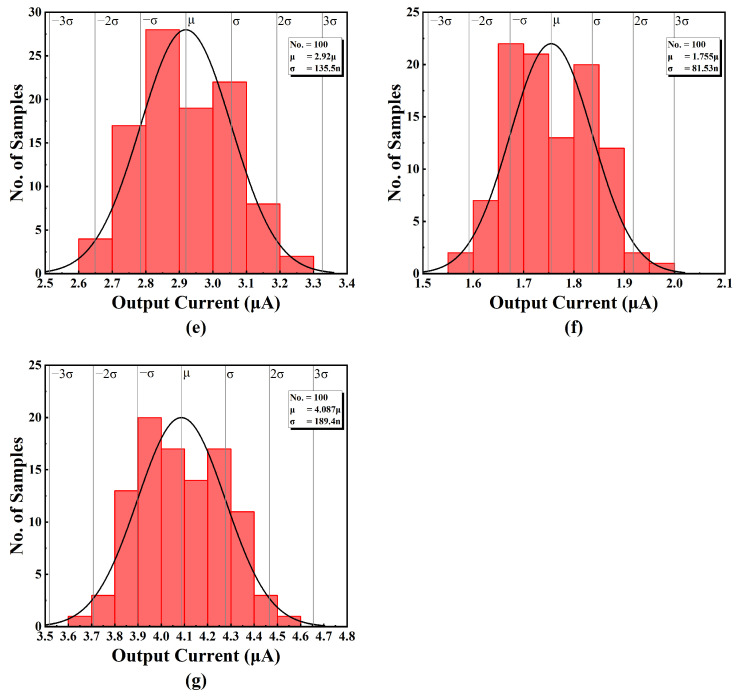
Monte-Carlo simulation result of (**a**) output current 1, (**b**) output current 2, (**c**) output current 3, (**d**) output current 4, (**e**) output current 5, (**f**) output current 6, and (**g**) output current 7.

Monte-Carlo simulations have bene used to evaluate the accuracy of the output currents under process variations and mismatch, which are presented in Figure 18. These Monte-Carlo simulations were performed at *V_DD_* = 0.45 V and the input DC voltage *V_IN_* = *V_DD_*/2 = 0.225 V.

The results are summarized in Table 3, showing the mean and the standard deviation of seven different output current levels, as well as the sensitivity of the respective output currents. It has been confirmed that the variation is around a few percentage points, which is acceptable in the context of process variation. Due to the programmable feature in this design, the variation can be compensated for by tuning in applications that require higher precision.

**Figure 19:**
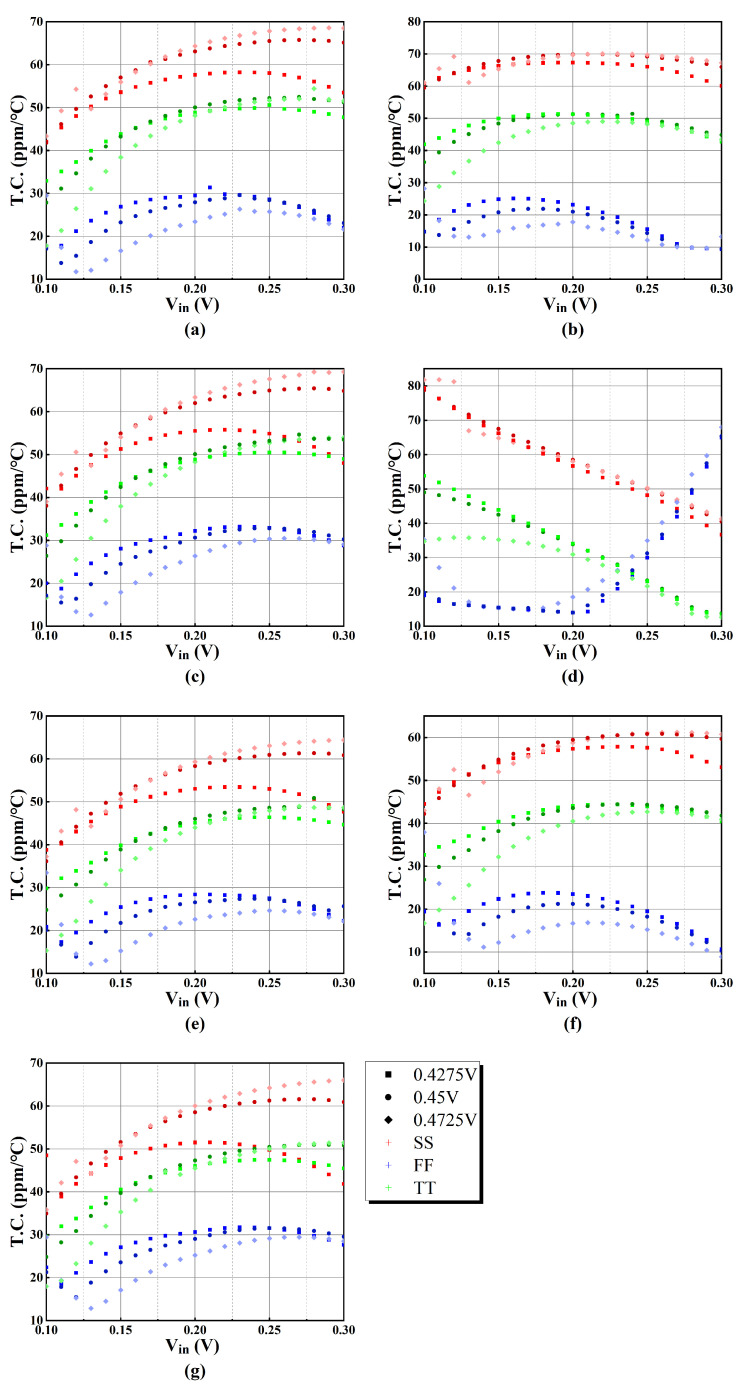
T.C. in PVT simulation of (**a**) output current 1, (**b**) output current 2, (**c**) output current 3, (**d**) output current 4, (**e**) output current 5, (**f**) output current 6, and (**g**) output current 7.

For each of the seven sets of output currents, their temperature coefficients at three different process corners are shown in Figure 19, where the temperature range is −20–80 °C.

The simulation results indicate that the T.C. ranges from 13.80 ppm/°C to 54.68 ppm/°C at a 0.45 V supply voltage and TT process corner. Considering TT, SS, and FF process corners, the T.C. ranges from 9.43 ppm/°C to 78.78 ppm/°C at 0.45 V supply voltage. When the voltage varies by ±5%, the T.C. ranges from 12.60 ppm/°C to 62.11 ppm/°C at the TT corner. The maximum and minimum values of T.C. are shown in Table 4. It can be observed that the variation in T.C. maintains reasonable values regardless of supply voltages and different corner variations.

**Figure 20:**
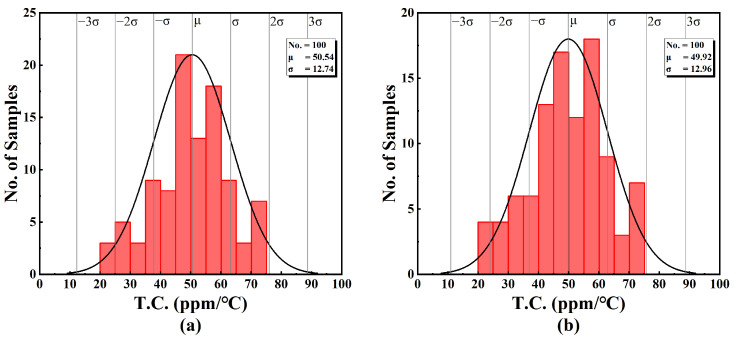

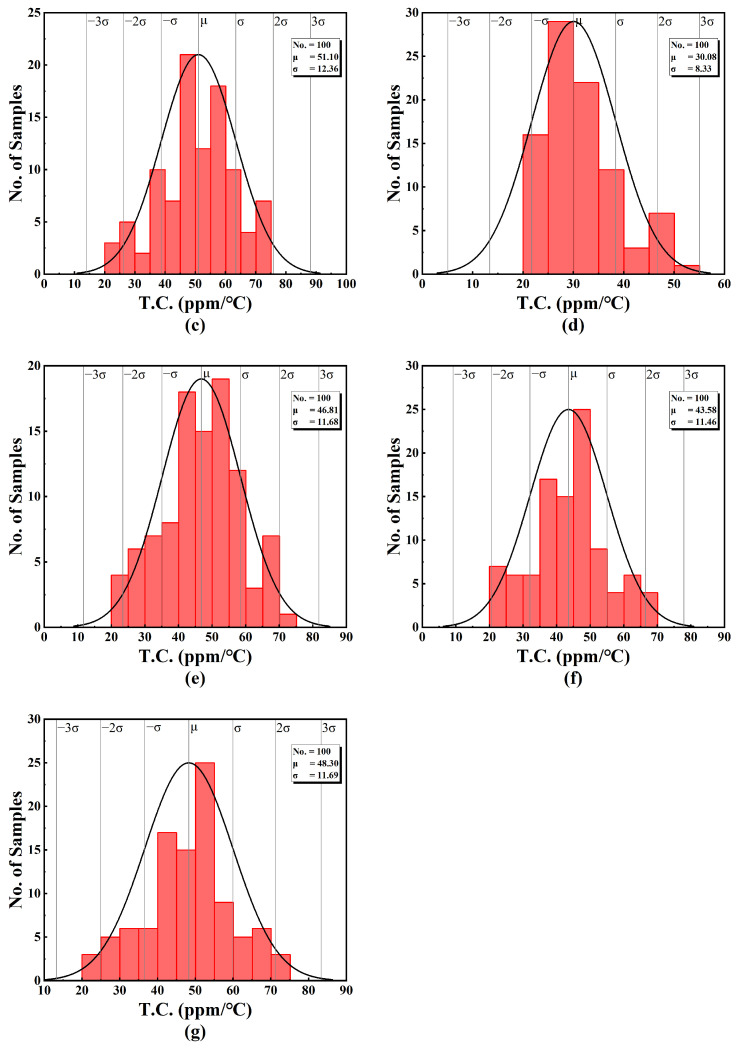
T.C. in Monte-Carlo simulation of (**a**) output current 1, (**b**) output current 2, (**c**) output current 3, (**d**) output current 4, (**e**) output current 5, (**f**) output current 6 and (**g**) output current 7.

In order to observe the change in T.C. under process mismatch and variations in statistical approach, seven sets of Monte-Carlo simulations, with each comprising 100 random sample points, are illustrated in Figure 20. These Monte-Carlo simulations are performed at *V_DD_* = 0.45 V, and the input DC voltage *V_IN_* = *V_DD_*/2 = 0.225 V.

The results show that the mean value of the temperature constant is less than 52 ppm/°C, with a standard deviation of less than 13 ppm/°C. The obtained statistical performance parameters are listed in Table 5.

**Figure 21:**
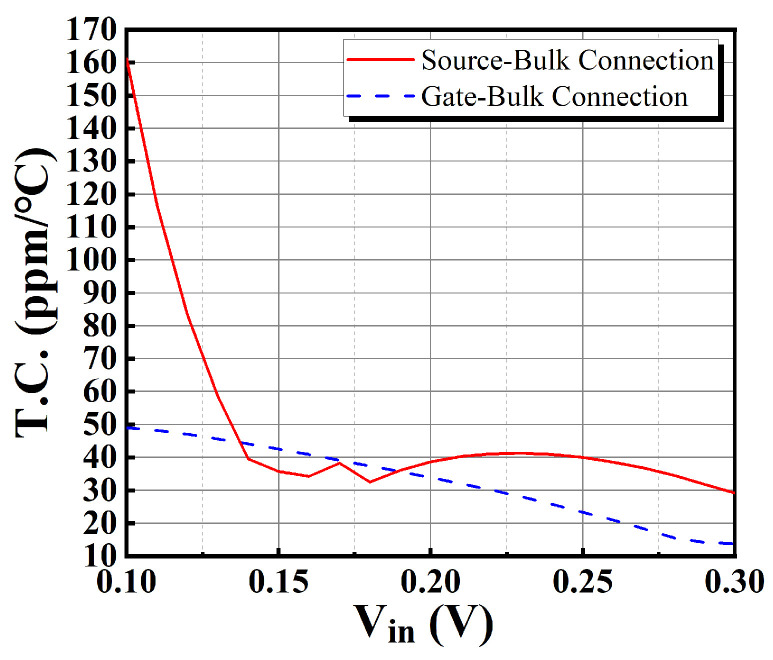
Comparison of T.C. of two different bulk connections.

For the benefits of using gate-bulk connections in level shifters, a comparison of the temperature coefficients of the output currents using gate-bulk connection and source-bulk connection is carried out at a supply voltage of 0.45 V and the TT process corner. This comparison is shown in Figure 21. The use of gate-bulk connection exhibits smaller and more stable temperature coefficients. Thus, the use of gate-bulk connections in level shifters helps optimize the temperature coefficient of the output current.

**Figure 22:**
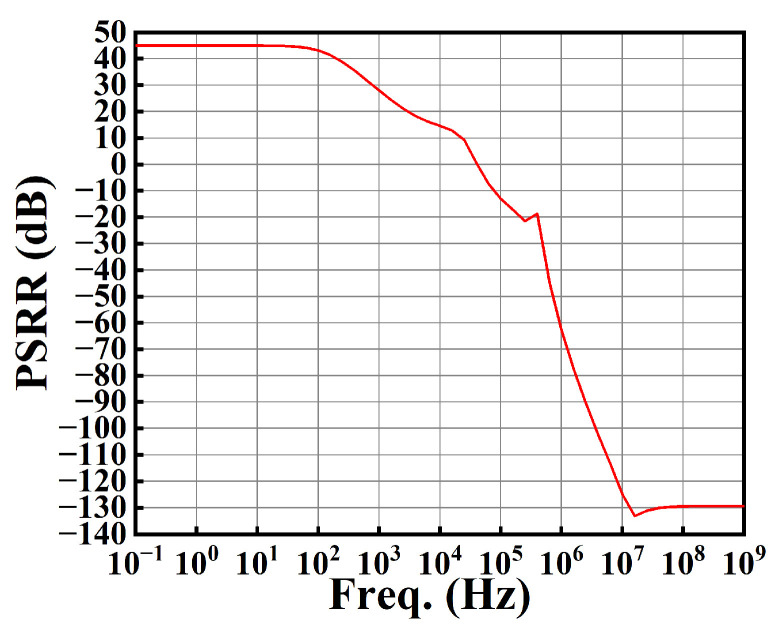
The plot of PSRR against frequency.

To evaluate the PSRR, the resistive test load is connected in parallel with *C*_4_ = 100 pF in Figure 6. From Figure 22, we see that the PSRR at low frequency is 44.96 dB.

**Figure 23:**
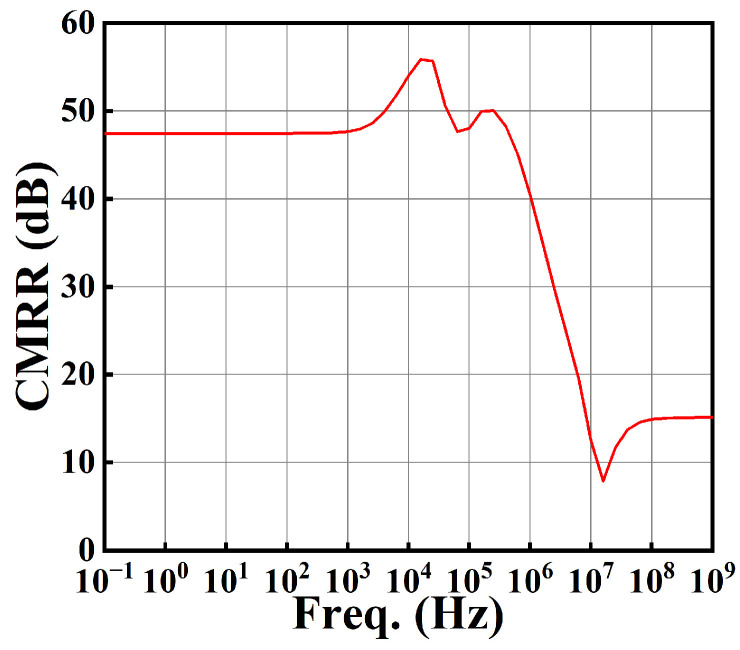
The plot of CMRR against frequency.

The CMRR of this circuit is shown in Figure 23. From Figure 23, we see that the CMRR at low frequency is 47.44 dB.

**Figure 24:**
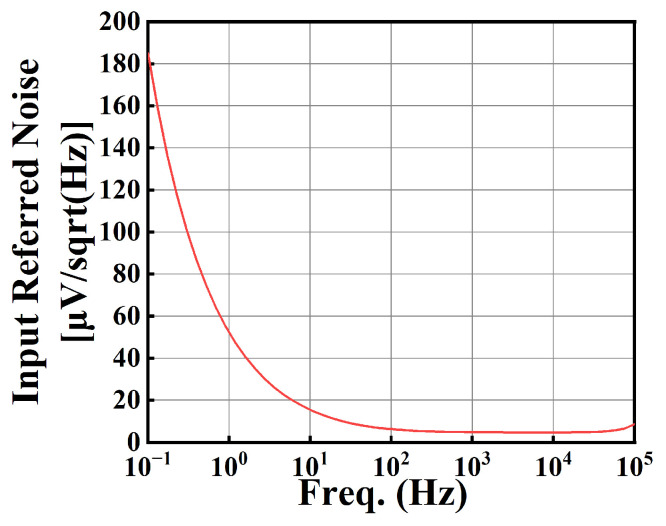
The input-referred noise spectrum.

Figure 24 illustrates the input-referred noise spectrum. The input-referred noise is 4.93 µV/sqrt(Hz) at an input signal frequency of 1 kHz.

**Figure 25:**
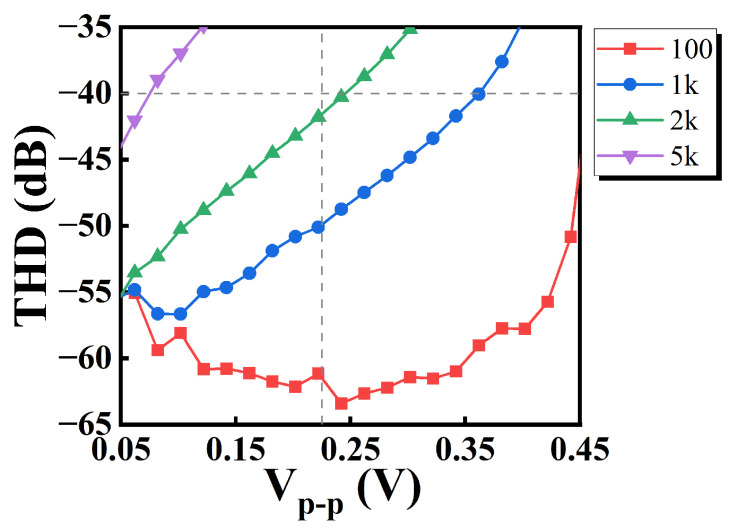
THD.

Figure 25 illustrates the relationship between THD and the peak-to-peak value of the input signal for different input frequencies. In this evaluation, the input signal has a DC quiescent bias voltage of 0.225 V, and the V-I Converter is powered at 0.45 V supply.

With *THD* = 1%, i.e., −40 dB as an upper limit, the input peak-to-peak value *V_pp_* can reach a maximum of 0.36 V when the input frequency is 1 kHz. When *V_pp_* is 0.225 V, the maximum input frequency can reach 2 kHz.

Table 6 shows a comparison in terms of performance with the previously reported designs. The figure of merit (FOM) is introduced to evaluate the quality of the work on a comparative basis. FOM is given by the following equation [28]:

(22) $$FOM=\frac{P_{w}\times Input\;Referred\;Noise}{Bandwidth}\mu W\cdot \left(\mu V/\sqrt{Hz}\right)/Hz$$

where *P_w_* is the power consumption of the system. The smaller the FOM, the better the overall performance of the circuit.

Referring to Table 6, we see that the conventional FOM does not account for temperature stability—a critical metric for precision analog circuits. The proposed circuit has demonstrated several key advantages for very low-voltage applications. First, this design achieves a temperature coefficient of 54.68 ppm/°C, which is 2× better than in [7] (113 ppm/°C) and 5× better than in [29] (276 ppm/°C). This ensures stable operation across a wide temperature range (−20 °C to 80 °C), making it suitable for precision applications in the context of temperature variation. Additionally, the ability to program output currents enhances its flexibility, enabling the circuit to be adapted to diverse scenarios, from biomedical sensors to energy harvesting systems. With a power consumption of only 0.36–2.76 μW, the circuit is ideal for battery-powered or energy-constrained applications, such as wearable devices and IoT nodes.

The proposed circuit also achieves a CMRR of 47.35 dB and a PSRR of 45.58 dB, which are comparable to those in the representative works. To optimize the trade-off between input-referred noise and temperature stability, a gate-bulk connection in the level shifter, which replaces the conventional source-bulk connection, is suggested. Although the circuit operates in the subthreshold region with Q-factor frequency compensation for stabilizing multiple gain stages in exchange for reduced bandwidth (34.45 kHz), the key objectives for attaining low-power consumption as well as the low temperature coefficient of the output currents have been confirmed. Future work can explore advanced compensation techniques to further improve bandwidth without compromising these critical advantages.

## 5. Conclusions

This paper describes a V-I Converter embedded with a three-stage OTA, which is based on a modified conventional structure. The circuit is designed using the TSMC 40 nm process and functions at a supply voltage of 0.45 V, enabling operation in the subthreshold region. The circuit has a programmable resistor array for a wide range of output currents when compared to previous designs, and guarantees a low temperature coefficient when pushing for the precision requirement. The low temperature coefficient is a critical parameter for very low-voltage cells. In addition, it has achieved lower power consumption and reasonable PSRR and CMRR. Therefore, the design can be adapted to a variety of application scenarios, and is useful for low-voltage, low-power analog signal processing and sensor signal processing applications. Compared to the previously reported designs, this design has lower bandwidth, which is due to the complicated frequency compensation allowed by its three-stage structure. This results in a restricted operating frequency range, leading to one of the limitations of the design. To this end, the design of the V-I Converter with low temperate coefficient awareness gives the extra benefit of larger operation headroom in the context of temperature variation.

## Figures and Tables

**Table 1:** Types and sizes of devices of proposed V-I Converter.

Device	Type	Size (W/L)	Device	Type	Size (W/L)
*M_B_*_1_	pch_lvt	10 μm/1 μm	*M*_9_	pch_lvt	840 μm/10 μm
*M_B_*_2_	pch_lvt	2 μm/1 μm	*R_B_*_1_	rppolywo	4 MΩ
*M_B_*_3_	pch_lvt	50.8 μm/20 μm	*R_B_*_3_	rppolywo	4.5 MΩ
*M_B_*_4_ *,_S_*_1_	pch_lvt	50 μm/20 μm	*R*_1_–*R*_4_	pch_lvt	600 nm/110 nm
*M_B_*_5__,*B*6_	nch_lvt	200 nm/6 μm	*R_array_*_1*a*_	rppoly	7.25 kΩ
*M_B_*_13_	nch	120 nm/1 μm	*R_array_*_2*a*_	rppoly	14.5 kΩ
*M_B_*_14_	nch	5 μm/1 μm	*R_array_*_4*a*_	rppoly	24.5 kΩ
*M_B_*_15__,*B*16_	pch_lvt	28 μm/1 μm	*R_array_*_1*b*_	rppolywo	89 kΩ
*M_B_*_17_	nch_lvt	2.5 μm/1 μm	*R_array_*_2*b*_	rppolywo	178 kΩ
*M_B_*_18_	nch_lvt	12 μm/1 μm	*R_array_*_4*b*_	rppolywo	360.5 kΩ
*M_S_*_2_	pch_lvt	19 μm/200 nm	*C_B_*_1_	cap	10 pF
*M*_0_	nch_lvt	30 μm/1 μm	*C_B_*_2_	cap	10 pF
*M*_1__,2_	nch_lvt	50 μm/1 μm	*C_B_*_5__,*B*6_	cap	10 pF
*M*_3__,4_	pch_lvt	8 μm/1 μm	*C*_1_	cap	1 pF
*M*_5__,8_	pch_lvt	70 μm/200 nm	*C*_2_	cap	5 pF
*M*_6__,7_	nch_lvt	6 μm/1 μm	*C*_3_	cap	10 pF

**Table 2:** Stability of OTA_o_ in PVT simulation.

V_DD_ (V)	Process Corner	DC Gain (dB)	PM (°)	BW (Hz)	UGB (kHz)
0.4275	TT ^1^	66.6	82.29	19.95	65.45
	SS ^2^	67.75	79.06	13.94	51.58
	FF ^3^	65.12	85.52	30.96	86.37
0.45	TT	67.97	79.12	17.69	70.04
	SS	69.2	75.34	12.23	55.13
	FF	66.53	82.76	27.43	93.07
0.4725	TT	68.94	75.39	16.42	74.99
	SS	70.2	71.08	11.29	58.84
	FF	67.49	79.55	25.45	100.1

^1^ Typical-Typical corner, representing nominal N transistors and nominal P transistors. ^2^ Slow-Slow corner, representing slow N transistors and slow P transistors. ^3^ Fast-Fast corner, representing fast N transistors and fast P transistors.

**Table 3:** Output currents in Monte-Carlo simulation.

	1	2	3	4	5	6	7
μ (μA)	2.335	1.17	3.503	0.586	2.92	1.755	4.087
σ (nA)	107.9	54.09	162.1	27.5	135.5	81.53	189.4
Sensitivity of Output currents [(σ/μ)%]	4.62	4.62	4.63	4.69	4.64	4.65	4.63

**Table 4:** T.C. summary.

V_DD_(V)	0.4275		0.45		0.4725		Overall	
T.C. (ppm/°C)	Min.	Max.	Min.	Max.	Min.	Max.	Min.	Max.
TT ^1^	13.56	53.83	13.80	54.68	12.60	62.11	12.60	62.11
SS ^2^	36.72	79.48	34.97	78.78	35.88	81.85	34.97	81.85
FF ^3^	9.33	64.95	9.43	65.39	8.88	68.03	8.88	68.03
Min.	9.33		9.43		8.88		NA ^4^	
Max.	79.48		78.78		81.85		NA	

^1^ Typical-Typical corner, representing nominal N transistors and nominal P transistors. ^2^ Slow-Slow corner, representing slow N transistors and slow P transistors. ^3^ Fast-Fast corner, representing fast N transistors and fast P transistors. ^4^ NA means Not-Applicable.

**Table 5:** T.C. in Monte-Carlo simulation.

	1	2	3	4	5	6	7
μ (ppm/°C)	50.54	49.92	51.10	30.08	46.81	43.58	48.30
σ (ppm/°C)	12.74	12.96	12.36	8.33	11.68	11.46	11.69
Sensitivity of T.C. [(σ/μ)%]	25.20	25.96	24.19	27.69	24.95	26.30	24.20

**Table 6:** Simulated performance comparison with previously reported works.

	2001 [29]	2007 [14]	2013 [7]	2019 [30]	2020 [17]	This Work
Technology (μm)	NA ^1^	0.5	0.18	0.18	0.13	0.04
Supply voltage (V)	+1/−2	±1.5	1.2	0.3	±0.2	0.45
Input range (V)	NA	0–3	0–1.1	0–0.3	−0.1–0.1	0.1–0.3
Temperature range (°C)	0–70	NA	−40–120	NA	NA	−20–80
T.C. (ppm/°C)	276	NA	113	NA	NA	54.68
Power consumption (μW)	NA	3000	85	0.01–0.1	0.36	0.36–2.76
BW (Hz)	NA	90 M	14.1 M	50–334	1.1 M	34.45 k
PSRR (dB)	46.2	35/43	47.8	50.2	52	45.58
CMRR (dB)	NA	62	NA	54.9	70	47.35
Input referred noise [µV/sqrt(Hz)]	NA	1.7	0.26	1.33	0.99	4.93
THD (dB@V_pp_@kHz)	NA	−60@6@100	−44.2@1@100	−46@0.1@0.1	−41.61@0.1@10	−56.66@0.1@1
FOM {µW·[µV/sqrt(Hz)]/Hz}	NA	5.67 × 10^−7^	1.56 × 10^−6^	3.98 × 10^−4^	3.24 × 10^−7^	5.15 × 10^−5^

^1^ NA means Not-Applicable.

## Data Availability

Data are contained within the article.

## References

[1] Rakus M., Stopjakova V., Arbet D. Comparison of gate-driven and bulk-driven current mirror topologies. Proceedings of the 2016 IEEE 19th International Symposium on Design and Diagnostics of Electronic Circuits & Systems (DDECS).

[2] Grasso A.D., Marano D., Palumbo G., Pennisi S. (2015). Design Methodology of Subthreshold Three-Stage CMOS OTAs Suitable for Ultra-Low-Power Low-Area and High Driving Capability. IEEE Trans. Circuits Syst. I Regul. Pap..

[3] Ferreira L.H.C., Sonkusale S.R. (2014). A 60-dB Gain OTA Operating at 0.25-V Power Supply in 130-nm Digital CMOS Process. IEEE Trans. Circuits Syst. I Regul. Pap..

[4] Lo T.-Y., Hung C.-C. 1-V Linear CMOS Transconductor with -65dB THD innAno-Scale CMOS Technology. Proceedings of the 2007 IEEE International Symposium on Circuits and Systems (ISCAS).

[5] Colletta G.D., Ferreira L.H., Pimenta T.C. (2014). A 0.25-V 22-nS symmetrical bulk-driven OTA for low-frequency *G_m_* -C applications in 130-nm digital CMOS process. Analog Integr. Circuits Signal Process..

[6] Yang G.-Z. (2018). Implantable Sensors and Systems: From Theory to Practice.

[7] Azcona C., Calvo B., Celma S., Medrano N., Martinez P.A. (2013). Low-Voltage Low-Power CMOS Rail-to-Rail Voltage-to-Current Converters. IEEE Trans. Circuits Syst. I Regul. Pap..

[8] Jakusz J., Jendernalik W., Blakiewicz G., Kłosowski M., Szczepański S. (2020). A 1-nS 1-V Sub-1-µW Linear CMOS OTA with Rail-to-Rail Input for Hz-Band Sensory Interfaces. Sensors.

[9] Silva-Martinez J., Salcedo-Suner J. A CMOS automatic gain control for hearing aid devices. Proceedings of the 1998 IEEE International Symposium on Circuits and Systems (ISCAS).

[10] Li F., Yang H., Liu F., Yin T., Wang X. (2012). Dual-mode gain control for a 1 V CMOS hearing aid device with enhanced accuracy and energy-efficiency. Analog Integr. Circuits Signal Process..

[11] Wang Y., Cao R., Li C., Dean R.N. (2021). Concepts, Roadmaps and Challenges of Ovenized MEMS Gyroscopes: A Review. IEEE Sens. J..

[12] Singhal N., Sharma R.K. Design of 4.9 GHz Current starved VCO for PLL and CDR. Proceedings of the 2018 5th International Conference on Signal Processing and Integrated Networks (SPIN).

[13] Wang C.-C., Lee T.-J., Li C.-C., Hu R. (2006). An All-MOS High-Linearity Voltage-to-Frequency Converter Chip with 520-kHz/V Sensitivity. IEEE Trans. Circuits Syst. 2 Analog Digit. Signal Process..

[14] López-Martín A.J., Ramirez-Angulo J., Carvajal R.G. (2007). 1.5 V 3 mW CMOS V–I converter with 75 dB SFDR for 6 V pp input swings. Electron. Lett..

[15] Khateb F., Kulej T., Vlassis S. (2017). Extremely Low-Voltage Bulk-Driven Tunable Transconductor. Circuits Syst. Signal Process..

[16] Ghosh S., Bhadauria V. (2021). An ultra-low-power near rail-to-rail pseudo-differential subthreshold gate-driven OTA with improved small and large signal performances. Analog Integr. Circuits Signal Process..

[17] Rico-Aniles H.D., Ramirez-Angulo J., Lopez-Martin A.J., Carvajal R.G. (2020). 360 nW Gate-Driven Ultra-Low Voltage CMOS Linear Transconductor With 1 MHz Bandwidth and Wide Input Range. IEEE Trans. Circuits Syst. II Express Briefs.

[18] Delbruck, Mead C.A. Adaptive photoreceptor with wide dynamic range. Proceedings of the 1994 IEEE International Symposium on Circuits and Systems (ISCAS).

[19] Ramirez-Angulo J., Carvajal R.G., Galan J.A., Lopez-Martin A. (2006). A free but efficient low-voltage class-AB two-stage operational amplifier. IEEE Trans. Circuits Syst. II Express Briefs.

[20] Djekic D., Fantner G., Lips K., Ortmanns M., Anders J. (2018). A 0.1% THD, 1-M Ω to 1-G Ω Tunable, Temperature-Compensated Transimpedance Amplifier Using a Multi-Element Pseudo-Resistor. IEEE J. Solid-State Circuits.

[21] Benko P.L., Galeti M., Pereira C.F., Lucchi J.C., Giacomini R. (2016). Innovative approach for electrical characterisation of pseudo-resistors. Electron. Lett..

[22] Wang S., Lopez C.M., Ballini M., Van Helleputte N. (2018). Leakage compensation scheme for ultra-high-resistance pseudo-resistors in neural amplifiers. Electron. Lett..

[23] Lau S.K., Mok P.K.T., Leung K. (2007). A Low-Dropout Regulator for SoC With Q-Reduction. IEEE J. Solid-State Circuits.

[24] Leung K., Mok P.K.T. (2001). Analysis of multistage amplifier-frequency compensation. IEEE Trans. Circuits Syst. 1 Fundam. Theory Appl..

[25] Zhang J., Chan P.K. (2021). A CMOS PSR Enhancer with 87.3 mV PVT-Insensitive Dropout Voltage for Sensor Circuits. Sensors.

[26] Wang D., Tan X.L., Chan P.K. (2017). A 65-nm CMOS Constant Current Source With Reduced PVT Variation. IEEE Trans. Very Large Scale Integr. Syst..

[27] Cheng Y., Chan M., Hui K., Jeng M.-C., Liu Z., Huang J., Chen K., Chen J., Tu R., Ko P.K. (1995). BSIM3v3 Manual.

[28] Fan X., Gao F., Chan P.K. (2023). Design of a 0.5 V Chopper-Stabilized Differential Difference Amplifier for Analog Signal Processing Applications. Sensors.

[29] Comer D.T., Comer D.J. (2001). CMOS voltage to current converter for low voltage applications. Analog Integr. Circuits Signal Process..

[30] Khateb F., Kulej T., Akbari M., Steffan P. (2019). 0.3-V Bulk-DrivennAnopower OTA-C Integrator in 0.18 µm CMOS. Circuits Syst. Signal Process..

